# Heterologous expression of *MirMAN* enhances root development and salt tolerance in *Arabidopsis*

**DOI:** 10.3389/fpls.2023.1118548

**Published:** 2023-04-14

**Authors:** Juanjuan Xu, Caiyu Yang, Shangyao Ji, Hui Ma, Jingwei Lin, Hui Li, Shuisen Chen, Hai Xu, Ming Zhong

**Affiliations:** ^1^ Key Laboratory of Agricultural Biotechnology of Liaoning Province, College of Bioscience and Biotechnology, Shenyang Agricultural University, Shenyang, Liaoning, China; ^2^ Collaborative Innovation Center for Genetic Improvement and High Quality and Efficiency Production of Northeast Japonica Rice in China, Rice Research Institute, Shenyang Agricultural University, Shenyang, Liaoning, China

**Keywords:** *mannanase*, salt stress, antioxidant enzyme system, transgenic Arabidopsis, *Mirabilis jalapa* L

## Abstract

**Introduction:**

β-Mannanase is a plant cell wall remodeling enzyme involved in the breakdown of hemicellulose and plays an important role in growth by hydrolyzing the mannan-like polysaccharide, but its function in adaptation to salt stress has been less studied.

**Methods:**

Based on cloned the *mannanase (MAN)* gene from *Mirabilis jalapa* L., the study was carried out by heterologously expressing the gene in *Arabidopsis thaliana*, and then observing the plant phenotypes and measuring relevant physiological and biochemical indicators under 150 mM salt treatment.

**Results and discussion:**

The results indicate that MirMAN is a protein with a glycohydrolase-specific structural domain located in the cell wall. We first found that MirMAN reduced the susceptibility of transgenic *Arabidopsis thaliana* to high salt stress and increased the survival rate of plants by 38%. This was corroborated by the following significant changes, including the reduction in reactive oxygen species (ROS) levels, increase in antioxidant enzyme activity, accumulation of soluble sugars and increase of the expression level of RD29 in transgenic plants. We also found thatthe heterologous expression of *MirMAN* promoted root growth mainly by elongating the primary roots and increasing the density of lateral roots. Meanwhile, the expression of *ARF7, ARF19, LBD16* and *LBD29* was up-regulated in the transgenic plants, and the concentration of IAA in the roots was increased. Those results indicate that *MirMAN* is involved in the initiation of lateral root primordia in transgenic plants through the *IAA-ARF* signalling pathway. In conclusion, MirMAN improves plant salt tolerance not only by regulating ROS homeostasis, but also by promoting the development of lateral roots. Reflecting the potential of the *MirMAN* to promote root plastic development in adaptation to salt stress adversity.

## 1 Introduction

Soil salinity is one of the major issues limiting plant growth and agricultural production, affecting an estimated 50% of arable land in China by 2050 (Jiang et al., 2019; Lu et al., 2019). High salinity reduces water absorption and ultimately leads to osmotic stress in the plant cell. Moreover, root uptake of large amounts of Na^+^ and Cl^−^ can harm metabolic processes and reduce photosynthetic efficiency (Oka et al., 2013). Oxidative stress, mediated by reactive oxygen species (ROS), is typically accompanied by osmotic stress and ionic toxicity. The effective control of the oxidative damage induced by osmotic and toxic is still critical for plant tolerance to stress.

The root is the first plant organ to encounter salt stress and sense the salt signal. Root activity and biomass are the main indicators to assess the tolerance of plants to stress due to directly affecting plant growth (Li et al., 2018). Root cells respond to adverse environments and boost endogenous defense mechanisms, involving the enhanced processes of synthesis of compatible osmolytes, antioxidants, hormones, transporters, and regulatory proteins. The upregulated expression gene, the response to dehydration 29A (*AtRD29A*), regarded as an indicator of stress tolerance, performs a protective and regulatory mechanism in plants (Jiang et al., 2019). These physiological processes further influence root system conformation *via* distinct mechanisms, including control of cell cycle progression, cell size and shape, and cell wall biosynthesis (Zelm et al., 2020).

Root cell proliferation, elongation, and differentiation all involve cell wall remodeling. As we know, the hemicellulose of the cell wall consists of many different polysaccharides that play a significant role in the highly dynamic changes of the cell wall (Cosgrove, 2005; Mohnen, 2008; Moreira and Filho, 2008). Mannans are major components of polysaccharides present within the plant cell wall and endosperm and are highly difficult to degrade (Jin et al., 2019). The enzymatic cleavage of mannans is achieved by several glycoside hydrolase (GH) enzymes, which act on concert to deconstruct the β-1,4-mannan backbone, ultimately releasing d-mannose and/or mannan oligosaccharides (Wang et al., 2013; Malgas et al., 2015), including mannan endo-1,4-β-mannosidase (EC 3.2.1.25) and 1,4-β-d-mannanase (EC 3.2.1.78), commonly named β-mannanase (MAN) (Sak-Ubol et al., 2016). According to the Carbohydrate-Active enZYmes (CAZY) database, MAN belongs to the glycoside hydrolase family 5 (GH5), a multigene family (Guillon et al., 2012). Previous studies have shown that endo-β-1,4-mannanases regulates a variety of plant growth and developmental processes including embryogenesis, leaf formation, stem elongation, shoot branching, fruit development, pollen development, and lateral root emergence (Nonogaki et al., 2000; Peters and Tomos, 2000; Buckeridge, 2010; Konozy et al., 2012; Yan et al., 2012; Wang et al., 2013; Carrillo-Barral et al., 2018; Yamamoto, 2019; Zang et al., 2019).

*PtrMAN6* in poplar is specifically expressed in xylem duct cells and catalyzes hydrolysis for the production of mannan oligosaccharides (Zhao et al., 2013). It confirms that MAN coordinates xylem cell expansion and secondary cell wall deposition. It has been determined that *AtMAN3* gene plays an important role in the regulation of heavy metal Cd tolerance in *Arabidopsis* (Chen et al., 2015). Notably, *MAN2/6* gene expression significantly increased in *Carex rigescens* following salt treatment from transcriptome data and then may have contributed to salt tolerance in *C. rigescens* (Zhang et al., 2020). In plants, MAN participates in a large number of developmental processes. Nevertheless, its function in abiotic stress tolerance remains unknown.

*Mirabilis jalapa* L., a kind of ornamental plant, has a strong tolerance to petroleum hydrocarbon-contaminated soil and the ability to promote the degradation of pollutant organic molecules (Peng et al., 2009). In this study, we successfully isolated the β-mannanases gene (*MirMAN*); *MirMAN* encodes a protein of 411 amino acids that contains a domain showing 68.13% sequence homology to the glycosyl hydrolase motif with *Spinacia oleracea* (XP_021852790.1), but its exact biological function has not yet been characterized. Complete loss of *MirMAN* function in plants suggests that *MirMAN* may be important for cell wall development and plant-related defense systems following salt stress. Accordingly, we generated *Arabidopsis* plants by heterologous expression of *MirMAN* and compared phenotypic features and responses to salt stress with wild-type plants. Then, we identified the role of *MirMAN* in responding to salt stress with an evaluation of the antioxidant status, soluble sugar accumulation, and lipid peroxidation, as well as an analysis of the expression levels of stress resistance-related genes. These results provide important new insights into the function of *MirMAN* gene in plant stress tolerance.

## 2 Materials and methods

### 2.1 Total RNA extraction and PCR amplification of *MirMAN* gene

*M. jalapa* RNA was extracted from plants grown with petroleum contamination for 45 days using the RNAprep Pure Plant Total RNA Extraction Kit (TianGen, Beijing, China) following the manufacturer’s instructions. After detection by 1% gel electrophoresis, the total RNA was first treated to remove the genomic DNA and then transferred into the first strand of cDNA using the PrimeScript RT reagent kit with gDNA Eraser (TaKaRa, Dalian, China) according to the manufacturer’s instructions. The first strand of synthesized cDNA was used as a template. The 2× PCR mix (TaKaRa, Dalian, China) was used to perform the PCR (**Supplementary Table 1**). The amplified product was recovered, directly ligated to the pMD-19T vector using the TA cloning method according to the operating instruction of pMD™ 19-T Vector Cloning Kit (TaKaRa, Dalian, China), and then transformed into TOP 10 competent cells. The positive clones, identified by bacterial liquid PCR using the sequencing primers M13 (M13F: TGTAAAACGACGGCCAGT, M13R: CAGGAAACAGCTATGACC) in the pMD-19T vector, were sent to Biobios (Shanghai, China) for sequencing.

### 2.2 *MirMAN* gene bioinformatics analysis

The molecular mass and isoelectric point of *MirMAN* were performed with ExPASy (http://web.expasy.org). The sequences of *MAN* in *Arabidopsis thaliana*, *L. esculentum*, and *Brachypodium distachyon* were obtained from the National Center for Biotechnology Information (NCBI) database (https://blast.ncbi.nlm.nih.gov/Blast). The sequence secondary and tertiary structures were predicted using SOPMA (https://npsa-prabi.ibcp.fr/cgi-bin/npsa_automat.pl?page=/NPSA/npsa_sopma.html) and SWISSMODEL (https://www.swissmodel.expasy.org/interactive/DTDUCp/models/), respectively. The sequence was analyzed for potential nuclear localization signals using cNLS Mapper (http://nls-mapper.iab.keio.ac.jp/cgi-bin/NLS_Mapper_form.cgi), transmembrane regions using TMHMM (Services- DTU Health Tech), and signal peptides using seqNLS (http://mleg.cse.sc.edu/seqNLS/). The predicted amino acid sequences of *MirMAN* and homologous genes were compared using DNAMAN, and a molecular phylogenetic tree was constructed using the neighbor-joining method in MEGA ver. 7.0.

### 2.3 Subcellular localization of *MirMAN:* GFP

Primer 5.0 software was used to design the primers pCAMBIA1303-*MirMAN*-F: GAAGATCTAAAGAAAGAAAAAATGAAAATAAAT and pCAMBIA1303-*MirMAN*-R: CGGACTAGTTCACCTCCTTAACTTTCTAATAATCCTA. With the use of the double-enzyme (*BgI*II and *Bam*HI) digestion method, the target gene was inserted into the plant overexpression vector pCAMBIA1303 to construct the pCAMBIA1303-*MirMAN :* GFP vector. The recombinant plasmid and PAD62 plasmid were transformed into competent *Agrobacterium tumefaciens* GV3101 cells, which were injected into tobacco leaves. Plants were grown in the dark for 1 day and then transferred to long-day conditions (16-h light/8-h dark) for 2 days. The leaf epidermal cells were incubated in 10% sucrose solution for 10 min to induce plasmolysis. The pCAMBIA1303-35S-*MirMAN :* GFP protein localization was observed under a laser confocal microscope (Leica, TCSSP8, Wetzlar, Germany).

### 2.4 Genetic transformation and identification of *MirMAN* overexpression transgenic lines

*Agrobacterium* LBA4404 containing the recombinant pCAMBIA1303-*MirMAN* overexpression vector was transfected into *Arabidopsis* Col-0 inflorescences, and the infected mature seeds (T0) were collected and cultured on 0.5× Murashige and Skoog (MS) (containing 20 mg/L of hygromycin) solid medium to screen for positive transformants. The *Arabidopsis* seedlings of various lines capable of rooting on the hygromycin-containing medium were identified by leaf PCR with a 2× PCR mix (Aidlab, Beijing, China) and the detection primers. A total of 33 transgenic lines with positive PCR results were selected for subsequent experiments. PCR conditions were 35 cycles of amplification (98°C for 10 s, 55°C for 5 s, and 72°C for 30 s). The total RNA was isolated from the transgenic *Arabidopsis* lines OE#1-33 and wild-type *Arabidopsis* using the RNA plant Plus reagent (TianGen, Beijing, China) based on the manufacturer’s instructions. The quantitative PCR (qPCR) was carried out using a FastKing RT Kit with gDNase (KR116, TianGen, Beijing, China) and SYBR Green qPCR Mix (Monad, Suzhou, China) according to the manufacturer’s protocols. qPCR was performed using Monad Selected q225MX (Monad Biotech Co., Ltd., Suzhou, China) real-time detection system. We used the reference gene *AtACTIN2* as endogenous controls for *Arabidopsis* (**Supplementary Table 1**). Three replicate reactions were routinely performed per sample.

### 2.5 Western blotting assays

For the Western blotting test of the *Arabidopsis* leaves, the protein content was measured using the method of Bradford (1976). Proteins were separated according to molecular weight by sodium dodecyl sulfate–polyacrylamide gel electrophoresis (SDS-PAGE) on a 10%–14% gradient gel and then transferred to an immobilon-P polyvinylidene difluoride (PVDF) membrane (Solarbio, Beijing, China). Proteins were conventionally detected with the MirMAN antibody (custom-made by GenScript, Piscataway, NJ, USA). Further, an anti-rabbit horseradish peroxidase (HRP) antibody was used as a secondary antibody. The blots were incubated with ECL Western Blotting Substrate (Solarbio, Beijing, China) and imaged using a Bio-Rad image analyzer (Bio-Rad, Hercules, CA, USA).

### 2.6 Morphological characterization of plant

Surface-sterilized seeds were plated on 0.5× MS medium with 1% sucrose and 1% agar (pH 5.7), and the plates were incubated in the dark at 4°C for 3 days. Then, plants were grown in the artificial growing room at a temperature of 22°C, 16-h light and 8-h dark (60% humidity and 120 μmol m^–2^ s^–1^ during a 16-h daily light period). We conducted digital images of root system morphology using a desktop scanner and determined the root length and the number of tips, forks, and crossings by using WinRHIZO. The seedlings were grown on 0.5× MS medium for 4 days, and then the agar plates were rotated 90°, causing the gravistimulus for 48 h. Data were compiled from three independent experiments with each line. Methods for the determination of IAA contents in the seedlings by mass spectrometry were described (Jia et al., 2020).

### 2.7 Salt stress treatment and physiological index determination of transgenic plants

For salt stress treatment, wild-type (WT) *Arabidopsis* and transgenic lines were planted in the same black plastic pots in the soil, and the plants were grown in a greenhouse. The 3-week-old seedlings in soil were irrigated with water and 150 mM of NaCl solution every 3 days and sampled at 0, 3, 5, and 7 days. Soil-grown *Arabidopsis* plants that were 24 days old were continuously watered with or without 150 mM of NaCl for 7 days, followed by an analysis of the survival rate after recovery for 1 week.

Catalase (CAT) activity was assayed on the basis of its ability to decompose H_2_O_2_ and measured at 240 nm. Superoxide dismutase (SOD) activity was determined by measuring its ability to inhibit the reduction of nitro blue tetrazolium chloride (NBT). Peroxidase (POD) activity was measured as the oxidation of guaiacol in the presence of H_2_O_2_, during which tetraguaiacol was formed. Lipid peroxidation in the plants was measured by the thiobarbituric acid-reacting substance and was expressed in terms of malondialdehyde (MDA) content. Diaminobenzidine (DAB) and NBT staining were used to confirm the detection of ${\text{O}}_{2}^{\;-}$ and H_2_O_2_, respectively. Glutathione (GSH) levels were measured according to the instructions supplied with the GSH Assay Kit (Suzhou Comin Biotechnology, Suzhou, China). Total soluble sugar was assayed by the anthrone method, which is widely used for the determination of soluble sugars. In brief, approximately 2 mL of the anthrone chemical reagent was added to 1 mL of the sample and then measured at 630 nm, and total soluble sugar content was calculated. Endo-β-mannanase activity was measured by a spectrophotometric method (Determination of activity β-mannanase as feed additives—Spectrophotometric method, GB/T 36861-2018, China). Briefly, the reducing oligosaccharides and monosaccharides can react with the 3,5-dinitrosalicylic acid reagent in a boiling water bath to yield a color change indicative of mannose hydrolysis. Each measurement was repeated at least three times, with data collection repeated at least three times.

### 2.8 Statistical analysis

All the data were presented as averaged values of three independent replicates. Statistical analyses of the data were carried out using Student’s *t*-tests, and multiple comparisons of means were analyzed using Tukey’s test. Differences were considered statistically significant at *p*< 0.05.

## 3 Results

### 3.1 Cloning and characterization of *MirMAN* in *Mirabilis jalapa*

**Figure 1:**
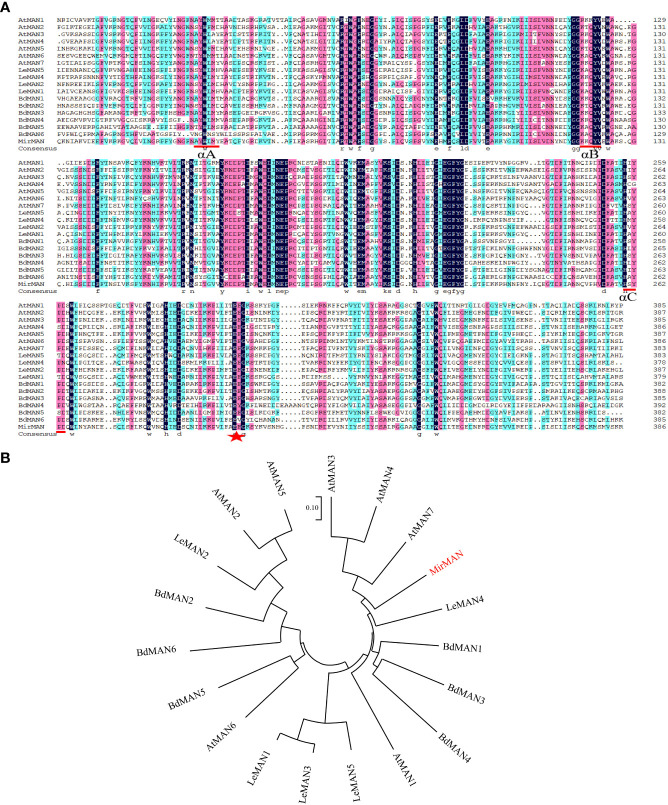
Alignment of sequences and phylogenetic tree of MirMAN proteins. **(A)** Alignment of sequences of MirMAN proteins and homologs from others species. Blue inverted triangles indicate signal peptide sites, red lines indicate the MAN conserved sequence, and red star indicates the catalytic glutamate residue structural motif. Multiple sequence comparisons showed that MirMAN was 46.96%, 45.85%, 43.99%, 42.65%, 42.31%, 41.79%, 39.72%, 39.15%, 39.08%, 35.71%, 35.03%, 34.98%, 34.57%, 34.41%, 33.64%, 31.98%, 31.57%, and 29.13%, consistent with LeMAN1, AtMAN7, LeMAN4, AtMAN3, AtMAN1, AtMAN4, LeMAN5, BdMAN1, LeMAN3, AtMAN6, BdMAN5, BdMAN2, AtMAN5, AtMAN2, BdMAN3, LeMAN2, BdMAN4 and AtMAN6, respectively. **(B)** Phylogenetic tree of MirMAN proteins from *Mirabilis jalapa* and other species. At, *Arabidopsis thaliana*; Bd, *Brachypodium distachyon*; Le, *Lycopersicon esculentum*.

The coding sequence of *MirMAN* was predicted from transcriptome sequencing results (data not published). The open reading frame (ORF) sequence of *MirMAN* is 1,233 bp long and contains 411 amino acids, with a predicted molecular weight of 47.08 kDa and an isoelectric point of 7.16 (pI 7.16). The calculated instability coefficient and hydrophobicity are 38.81, and −0.46, respectively, which are indicative of stable hydrophilic protein. Multiple sequence comparisons showed that MirMAN was 46.96%, 45.85%, 43.99%, 42.65%, and 42.31% consistent with LeMAN1, AtMAN7, LeMAN4, AtMAN3, and AtMAN1, respectively (**Figure 1**). In the secondary structure of MirMAN, α-helix accounted for 38.2%, β-sheets for 10.7%, extension chain for 20.9%, and random structures for 30.17%. Computational analysis predicted that *MirMAN* folds in a typical (β/α)8-TIM barrel structure, with multiple β-sheets forming a hydrophobic domain (**Supplementary Figure 2**).

### 3.2 Subcellular localization of MirMAN protein

**Figure 2:**
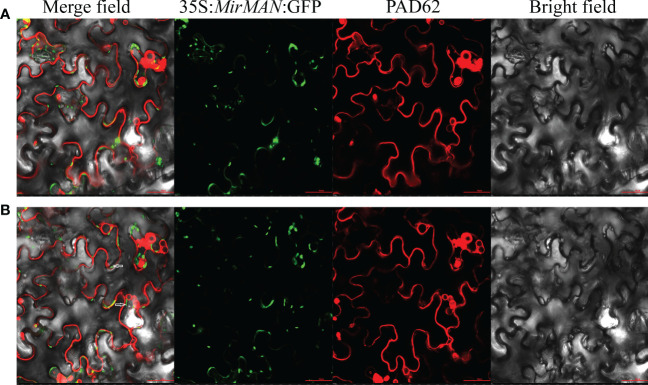
Subcellular localization of MirMAN. **(A)** Subcellular localization of 35S:*MirMAN:* GFP in tobacco cells. **(B)** Subcellular localization of 35S:*MirMAN:* GFP following 30% sucrose treatment. Bar = 50 μm. After plasmolysis, GFP signal observed in the cell wall is indicated by white arrows.

The marker for plasma membrane (PM), PAD62-mcherry, was co-expressed with pCAMBIA1303-35S-MirMAN-GFP by *Agrobacterium*-mediated transient expression in *Nicotiana benthamiana* leaves. After 16 h of agroinfiltration, we observed fluorescence in the green channel with a confocal laser scanning microscope and compared its position. Red PAD62 fluoresced in membrane epithelial cells (**Figure 2A**). pCAMBIA1303-35S-MirMAN-GFP green fluorescence was clearly distributed in a dotted pattern in the cell (**Figure 2A**). To investigate whether these signals were outside or inside the membrane, 30% sucrose was added to induce plasmolysis of *N. benthamiana* leaf cells, and images were acquired using a confocal microscope. Upon plasmolysis, the pCAMBIA1303-35S-MirMAN-GFP signal remained predominantly associated with the cell wall, although the PAD62-labeled PM retracted from the cell wall (**Figure 2B**).

### 3.3 Generation of transgenic *Arabidopsis* lines

**Figure 3:**
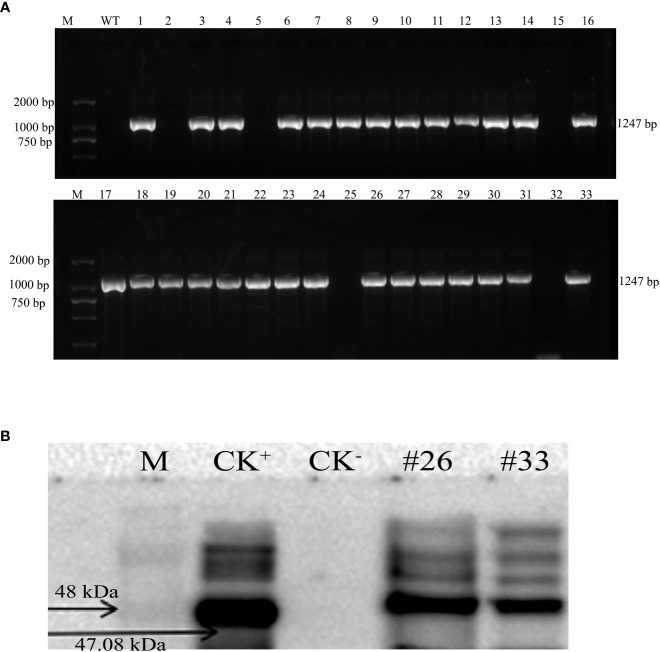
Characterization of recombinant *MirMAN* gene in *Arabidopsis.* **(A)** Cloning of *MirMAN* gene and PCR identification of T2 transgenic *Arabidopsis.* **(B)** The specific antibody was used for Western blotting. **(A)** M: DL marker 2,000; WT: wild type (negative control); the others are the 33 transgenic lines. **(B)** M: marker; CK^+^, positive control; CK^−^, negative control; the others are the transgenic lines.

The *MirMAN* ORF sequence was conducted into a plant binary vector control of the constitutive CaMV 35S promoter and transformed into *Arabidopsis* Columbia-0 (Col-0) plants. Thirty-three independent transgenic *Arabidopsis* lines were generated, their identity from 33 lines had already been confirmed by PCR analysis, and these lines were designated OE#1–33 (**Figure 3A**). Expression of MirMAN protein in the transgenic lines was demonstrated by Western blotting. **Figure 3B** shows the result of Western blotting after polyclonal MirMAN antibody detection. As shown in **Figure 3B**, a clear band was observed at approximately 47 kDa and was specifically precipitated by the antibody, but not in the control group. These results indicate that MirMAN has been successfully integrated into the *Arabidopsis* genome. Therefore, T3 mutants (#20, #26, and #33) were selected for further analysis. We identified *MirMAN* gene expression levels in T3 generation transgenic lines under normal conditions (**Supplementary Figure 1**).

### 3.4 Effects of root system architecture in *MirMAN* heterologous-expressing *Arabidopsis*

**Figure 4:**
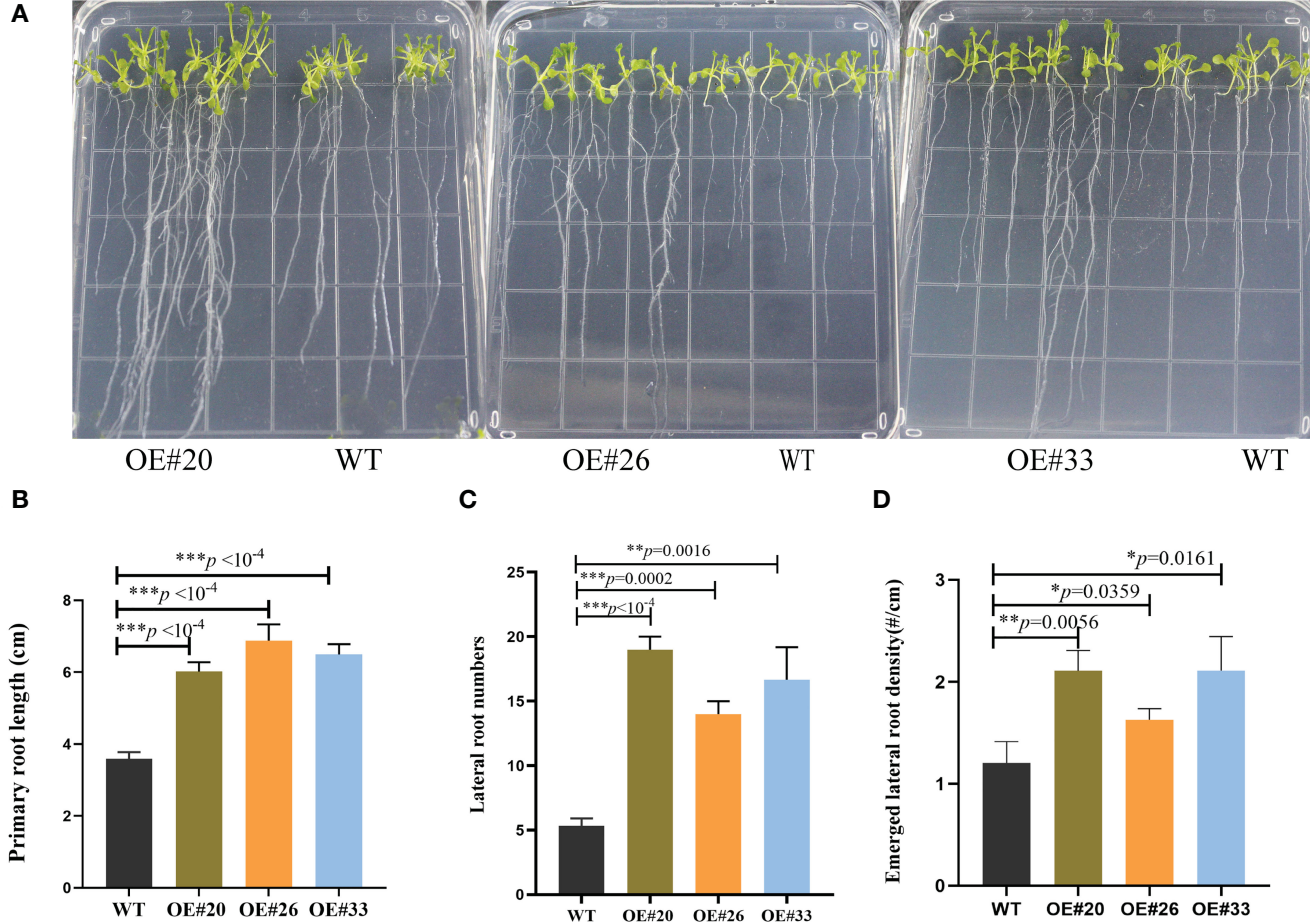
MirMAN regulation of root development on transgenic *Arabidopsis* plants. **(A)** The phenotypic root observation of wild type (WT) and *MirMAN-OE* lines. **(B)** Length of the primary root. **(C)** Number of lateral roots. **(D)** Density of lateral root primordia. The seedlings were cultured for 12 days. Data are means ± SD (n = 10). *p< 0.05, **p< 0.01, ***p< 0.001, independent-samples *t*-test.

**Figure 5:**
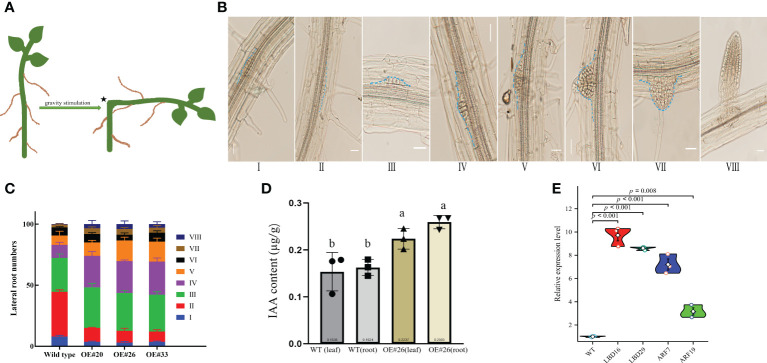
Comparison of lateral root density of wild type (WT) and OE lines *Arabidopsis* plants at various developmental stages. **(A)** Diagram showing a classic approach that was used to induce lateral root initiation and formation. Bending the horizontally placed primary root tip 90° and allowing the root tip to grow in the direction of gravity can induce the initiation and formation of lateral roots at the convex side of the bending site, as indicated by an asterisk. **(B)** Lateral root formation at different developmental stages detected from a root of single plant. **(C)** Measurements of the percentage of lateral root formation of WT and OE lines *Arabidopsis* at various developmental stages (*n* = 100). Measurements were carried out after gravity stimulation for 48 h. **(D)** IAA content in WT and OE#26 seedlings. **(E)** Expression level of *LED16*, *LBD29*, *ARF7*, and *ARF19* genes. At least three independent biological replicates were carried out, and consistent results were obtained. Results from one representative experiment are shown. Ten-day-old seedlings were used for analysis. Data shown represent average and SD (*n* = 100). Letters indicate the results for comparisons across groups using Tukey’s *post hoc* tests.

Compared to WT *Arabidopsis*, transgenic *Arabidopsis* lines #20/26/33 display an increased total number of lateral roots and primary root lengths in the 0.5× MS medium (**Figure 4**). Indeed, we noticed that the OE#20/26/33 lines with numerous lateral roots were up to 3.5–4-fold. To assess this possibility, we used gravitropic stimulation to synchronize lateral root primordia initiation in WT and OE#20/26/33 seedlings (**Figure 5A**). The period of the seedlings’ lateral root primordia emergence was defined as eight time periods, and their temporal developmental axis is shown in **Figure 5B**. We observed that transgenic *Arabidopsis* #20/26/33 lines display increased lateral root density and lateral root primordia per developmental stage III–VIII (**Figure 5C**). To better understand the relationship between IAA content and the numerous lateral roots, transgenic *Arabidopsis* lines’ IAA content was tested. IAA content was more accumulated in transgenic *Arabidopsis* line #26 (**Figure 5D**). We focused on the expression of root development-related genes, which was upregulated by 3–10-fold in transgenic lines compared to that of control plants, including *LBD16* (lateral organ boundaries domain), *LBD29*, *ARF7* (auxin response factor), and *ARF19* (**Figure 5E**).

### 3.5 Salt tolerance analysis of *MirMAN* heterologous-expressing transgenic *Arabidopsis*

**Figure 6:**
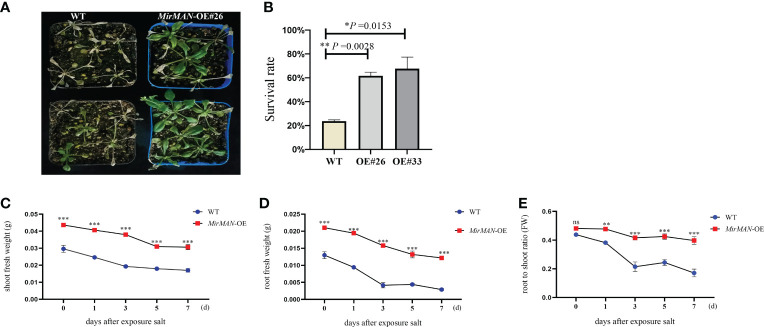
Effect of salt stress on *MirMAN-*overexpressed *Arabidopsis* plants. **(A)** Plants were grown on soil for 3 weeks and then exposed to 150 mM NaCl stress. **(B)** Survival rate under 150 mM NaCl stress. **(C)** Shoot fresh weight of plants. **(D)** Root fresh weight of plants. **(E)** Root-to-shoot ratio. *n* = 5. Error bars indicate ± SD. Student’s *t*-tests were used to compare the measurements of OE lines with those from wild type (WT). *p< 0.05, **p< 0.01, ***p< 0.001. ns represents no significance at *p* > 0.05.

In the comparison of phenotypic differences between the WT and the OE#26 plants under salt stress, the treatment of WT *Arabidopsis* with 150 mM of NaCl strongly inhibited root growth, and OE#26 showed less growth inhibition when treated with 150 mM of NaCl in soil (**Figure 6**). To determine salt tolerance in soil, we treated 24-day-old soil-grown plants with 150 mM of NaCl for additional 7 days, followed by recovery for 1 week. Most of the leaves of WT *Arabidopsis* gradually turned yellow, shrunk in size, and wilted, but the wilting symptoms of OE#26 plants were significantly slower than those of the control (**Figure 6A**). In addition, after rehydration, 61% of OE#26 plants and only 23.0% of control plants resumed their growth (**Figure 6B**). Salt stress significantly inhibited root and shoot fresh weight in WT and transgenic *Arabidopsis*. Specifically, the OE#26 plants showed a significantly higher root fresh weight, shoot fresh weight, and root/shoot ratio than those in WT plants under salt stress (*p* ≤ 0.05) (**Figures 6C, D**). While WT plants responded to salt stress by decreasing their root-to-shoot ratio, the ratio in transgenic *Arabidopsis* remained unchanged (**Figure 6E**).

### 3.6 *MirMAN* heterologous expression improves free radical scavenging activity and antioxidant status

**Figure 7:**
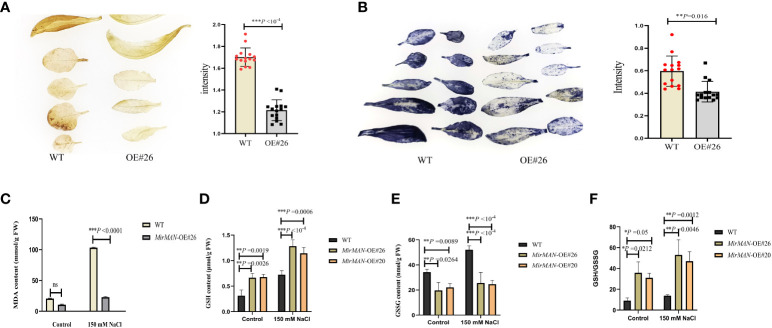
Evaluation of salt resistance in WT and OE lines *Arabidopsis* plants. **(A)** H_2_O_2_ contents. **(B)** ${\text{O}}_{2}^{\;-}$ contents. **(C)** MDA contents. **(D)** GSH contents. **(E)** GSSG contents. **(F)** GSH/GSSG. MDA, malondialdehyde; GSH, glutathione; GSSG, oxidized glutathione; WT, wild type. Error bars indicate ± SD. Student’s *t*-tests were used to compare the measurements of OE lines with those from WT. *p< 0.05, **p< 0.01, *** *p<* 0.001. ns represents no significance at *p* > 0.05.

**Figure 8:**
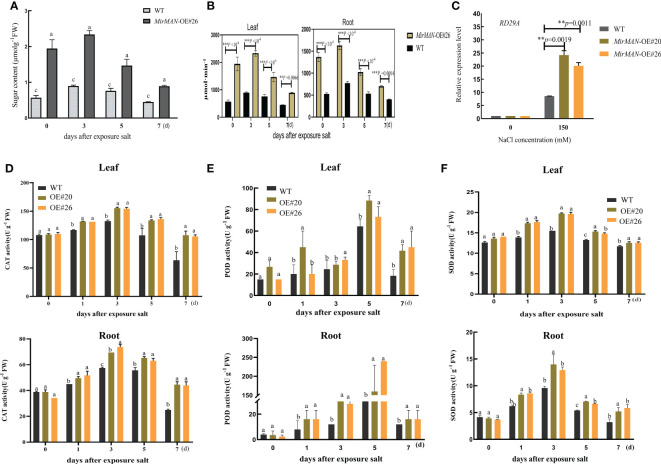
Salt resistance analysis of WT and transgenic lines of *Arabidopsis* plants. **(A)** Soluble sugar contents. **(B)** Endo-β-mannanase activity. **(C)** *RD29A* gene expressions after salt experiment. **(D)** CAT activity. **(E)** POD activity. **(F)** SOD activity. SOD, superoxide dismutase; POD, peroxidase; CAT, catalase; WT, wild type. Error bars indicate ± SD. Student’s *t*-tests were used to compare the measurements of OE lines with those from WT. **p< 0.01, *** *P* < 0.001. Letters indicate the results for comparisons across group using Tukey’s *post hoc* test.

We next tested the physiological alterations in WT and OE#20/26 plants under salt stress conditions. We also measured the H_2_O_2_ and ${\text{O}}_{2}^{\;-}$ characteristics by chemical staining and analyzed the H_2_O_2_ and ${\text{O}}_{2}^{\;-}$ contents of WT and OE#26 plants after salt treatment. We found that WT plants accumulated more H_2_O_2_ and ${\text{O}}_{2}^{\;-}$ than OE#26 plants (**Figures 7A, B**). Upon NaCl treatment, the average MDA content in WT plants was 4.49 times higher than in MirMAN heterologous expression plants (**Figure 7C**). The OE#20/26 plants increased the levels of GSH compared to the values of the WT *Arabidopsis* during the normal conditions (**Figure 7D**). Upon salt treatment, the values of GSH and GSH/GSSH in both the OE lines and WT plants increased significantly, with the OE lines exhibiting significantly higher levels than WT *Arabidopsis* (**Figures 7D, F**). The opposite was true for oxidized GSH (GSSG) concentrations, whose levels were 25.64 and 24.67 significantly lower for the OE lines compared to the WT (52.09), respectively (**Figure 7E**). In addition, GSH/GSSG values in the OE lines under salt stress increased to a greater extent compared to those in the WT *Arabidopsis* (**Figure 7F**). The total soluble sugar content in the OE#20/26 plants was 1.6 times that in the WT plants (**Figure 8A**). Under control conditions, total mannanase activity in both OE lines had significant increases compared to that in the WT plants (**Figure 8B**), showing that increased *MirMAN* overexpression resulted in more mannanase production. Moreover, salt stress increased the transcript levels of *RD29A* in transgenic plants by at least twice compared to those in WT plants (**Figure 8C**). Furthermore, our data show that *MirMAN* substantially increases CAT, POD, and SOD activities (**Figures 8D–F**).

## 4 Discussion

Although the orthologues of *MAN* genes have been identified in many plant species, such as *L. esculentum*, *B. distachyon*, and *Brassica rapa*, the influence of *MirMAN* on the regulation of growth remains almost unknown (Nonogaki et al., 2000; Iglesias-Fernández et al., 2011; González-Calle et al., 2015; Carrillo-Barral et al., 2018). Here, *MirMAN* was cloned from *M. jalapa* L., and its function of improving the growth and salt tolerance of plants was analyzed in *Arabidopsis*.

### 4.1 Characteristic and subcellular localization analyses of *MirMAN*

Sequence and phylogenetic analyses showed that *MirMAN* is homologous to *AtMAN7*, but its function has not been fully resolved (**Figure 1**). Here, we demonstrated that the heterologous expression of *MirMAN* can substantially alter relevant root development without abiotic stress in *Arabidopsis*. Like other MANs, *MirMAN* has a putative signal peptide and an active site with the expected conserved amino acids. The *MirMAN* is composed of a sole GH5 catalytic module without any attached carbohydrate-binding module (CBM). Judging from the subcellular distribution of *MirMAN :* GFP protein fluorescence signal in *Nicotiana tabacum* cells, *MirMAN* was localized to the cell wall (**Figure 2**). The subcellular localization of *MirMAN* on the cell wall might play an important role in cell-wall loosening, as many cell expansion requires primary cell-wall loosening and incorporation of newly synthesized cell-wall material.

### 4.2 Effects of heterologous expression of *MirMAN* on plant growth in *Arabidopsis*

Heterologous expression of *MirMAN* gene in *Arabidopsis* enhances plant growth. The effect of 35S: *MirMAN* transgenic plants on leaf development was particularly striking in rosette leaves. In *MirMAN* transgenic plants, the rosette leaves were large and elongated with increases in the compactness of the branches and leaves of the plants (**Supplementary Figure 3**). *MirMAN* causes a series of metabolic changes in transgenic plants, among which the increased cell growth rate is one of them. Moreover, the heterologous expression of *MirMAN* obviously changed root system architecture in transgenic plants, including longer primary roots and more numbers of lateral roots (**Figures 4**, **5**). This indicates that *MirMAN* ultimately affects lateral root formation at different developmental stages. Lateral roots are important not only for determining plants’ root system phenotypes in soil but also for absorbing water and nutrients (Lucob-Agustin et al., 2020). Several studies have set to demonstrate cell wall composition and extensibility mediated by cell wall modification enzymes necessary for root cell elongation, thereby controlling root elongation and, consequently, root development (Tabuchi and Matsumoto, 2001; Ma et al., 2004). In our study, the number of lateral roots of transgenic plants increased, which may enable them to store more water. Transgenic plants showed an enhanced survival capacity compared with the control plants in salt treatment.

Exogenous application of auxin or improved endogenous auxin synthesis leads to a significant increase in the number of lateral roots (Laskowski et al., 1995). In these experimental results, the increase of lateral roots of overexpression *MirMAN* is closely related to the increase of endogenous auxin (**Figure 5D**). It is speculated that there may mechanisms, as follows: 1) the increase of auxin concentration promoted the early arrival of auxin oscillation peak. After detection of the initial signal auxin, rhizosphere meristem cells rapidly select lateral root cells, accelerate lateral root primordium development, and further develop lateral root organs. 2) When the lateral root primordium breaks through the epidermis, the cell wall is more likely to change; that is, cells are more likely to detach and form new cells, thereby speeding up the overall lateral root development. 3) The polysaccharide composition of the cell wall changed. Mannanase cleaves mannan or mannan oligosaccharide, and its products mannan oligosaccharide and mannose are speculated to be involved in upstream signal control hormone signal, which may have a positive development feedback mechanism, but the relationship between them needs to be further proved.

Like other *MAN*s, C has putative glutamate catalytic residues for breaking the mannosidic bond (http://smart.embl-heidelberg.de/). Typical MAN proteins are composed of three helices: αA, αB, and αC (**Figure 1**). *MirMAN* is mainly predicted to control the extensibility of the cell wall by causing crosslinking or cleaving of cell wall polysaccharides and thus promote cell wall loosening and therefore growth. We speculated that *MirMAN* promotes hydrolysis of hemicellulose by endo-β-1,4-mannanases, thereby enhancing cell wall expansion. Endo-β-mannanase hydrolyzes mannan oligosaccharide or mannans to produce mannose and/or mannan oligosaccharide (Wang et al., 2013). The research found that galactoglucomannan oligosaccharide (GGMO) treatment, while promoting the primary root elongation and stimulating the emergence of lateral roots, influences *Zea mays* L. growth development (Kollárová et al., 2018). Previous studies have focused on the downstream effects of lateral root formation during root development, as glucose acts upstream of the BR signal transduction pathway to regulate the lateral root process by dose-dependently. Similarly, glucose also acts on the fiber elongation in cotton directly by BR signal transduction (Gupta et al., 2015; Li et al., 2022).

### 4.3 Heterologous expression *MirMAN* most likely leads to salt tolerance in *Arabidopsis*

Salt stress is dramatically imposing severe constraints on plant adaptation, crop productivity, and quality. The search for molecule regulation of plant stress responses and plant development remains a key priority in the fight against abiotic stress resistance. As plants are exposed to abiotic stresses, most of the root system architectures (RSAs) altered according to the environment contribute to tolerance and recovery, which involves gene expression of root cell wall remodeling (Motte and Beeckman, 2019; Zhang et al., 2019). Overexpression of *MirMAN* causes suitable root phenotypes in transgenic plants within salt stress, including main root elongation and promoting lateral root density (**Supplementary Figure 4**), which positively regulates the salt stress resistance. This positive effect may have partially offset the growth inhibition induced by salt stress in transgenic *Arabidopsis*.

Adversity can cause the overproduction of ROS and thereby affects oxidative stress, endoplasmic reticulum (ER) stress, and consequent damage to proteins and subcellular organelles in plants (Mittler et al., 2011). Several cellular mechanisms have been shown to counterbalance the production of ROS, including enzymatic and non-enzymatic pathways. In plants undergoing salt stress responses, antioxidant enzyme activities are important for suppressing ROS-induced oxidative stress. At 150 mM NaCl-treated groups, H_2_O_2_ and ${\text{O}}_{2}^{\;-}$ were overproduced in wild-type plants (**Figures 7A, B**). Considering SOD, CAT, and ascorbate peroxidase (APX) activity data in transgenic lines of *Arabidopsis* (**Figures 8D–F**), it is concluded that both antioxidant enzymes successfully eliminate the excess of H_2_O_2_ and ${\text{O}}_{2}^{\;-}$ , avoiding the oxidative stress damage caused by salt stress. By contrast, the heterologous expression in *MirMAN* plants shows a lower MDA content than in wild-type plants (**Figure 7C**). Similarly, glutathione is an endogenous antioxidant that exists in either reduced (GSH) or oxidized (GSSG) form that protects various cellular structures from oxidative damage. Under salt stress, the GSH levels of both WT and *MirMAN* transgenic lines of *Arabidopsis* were significantly upregulated, but the GSH level of OE#26 was more significant (**Figure 7D**), indicating that there are more GSH in the OE#26 against more oxidative stress. Also, in the transgenic plants, more glutathione was used for the reduction reaction, and less glutathione was produced by oxidation (**Figure 7E**). Glutathione accumulated in higher concentrations in *MirMAN* transgenic lines, which in turn helped plants to withstand oxidative stress mediated by the H_2_O_2_ and ${\text{O}}_{2}^{\;-}$ during episodes of salt stress. These results suggest that overexpression of *MirMAN* plants reduces the accumulation of oxidative H_2_O_2_ and ${\text{O}}_{2}^{\;-}$ by enhancing antioxidant defenses to oxidative stress. The *MirMAN* transgenic *Arabidopsis* lines have optimized an antioxidant defense system to increase salt tolerance. Soluble sugar fraction levels in these lines are significantly increased compared with wild-type levels (**Figure 8A**). Plants can use metabolites to reduce the osmotic potential of plants, which prevents the imbalance of ROS production and ensures water balance for the normal growth of plants under salt stress. The metabolites are bioactive molecules that play a key role in stress, but they are also osmotic regulators of many intracellular processes including preventing membrane fusion and stabilizing enzymes and other intracellular components to cope with salt stress. The relationship between these metabolites interacting with salt tolerance needs further studies (Ismail and Horie, 2017; Liu et al., 2021).

Moreover, we believe that the proposed root development strategy could also substantially improve their performance in salt stress and recovery efficiency. Studies showed that the BR signal transduction component *BES1* directly affects the expression of cell wall remodeling genes *XTH19* and *XTH23* as a positive regulator of salt stress, which promoted the development of lateral roots and thus improved the salt adaptability (Xu et al., 2020). Mannose acts as a signal molecule to regulate plant growth and development (Richterová-Kučerová et al., 2012). It suggests that these sugar molecules can specifically integrate various responses to endogenous and environmental signals to modulate root development in *Arabidopsis* (Valifard et al., 2021). Plant abiotic stress resistance is improved by the application of the small molecule sugar and interferes with the transcriptional dynamics of downstream signal transduction, indicating that it plays an important role in stress-related pathways. Most of the underlying molecular mechanisms remain unclear (Liu et al., 2021). Understanding how the *MirMAN* hydrolysate perceives, integrates, and responds to environmental signals is critical for improving plant resistance to stress and will be the focus of our future research efforts.

The experiment aimed to study the response mechanism of *MirMAN* to salt stress. Little is known about the physiological functions of these enzymes *in planta*. Although the causal relationship between hydrolysis products of mannanase and lateral root development is yet to be established by designing further experiments, this provides us a new insight into the accumulation of endo-β-mannanase in plants, which causes a change in plant growth and development under salt stress. It also provides further intriguing evidence suggesting that the cell wall remodeling gene may have widespread and as yet largely unrecognized functions in plant growth, development, and plant stress tolerance. At present, we cannot distinguish between direct effects (e.g., specific binding to a receptor) and indirect effects (e.g., hydrolysate). Nevertheless, elucidation of the mechanistic basis of these effects is likely to provide new insights into the factors that govern plant growth and stress tolerance.

**Figure 9:**
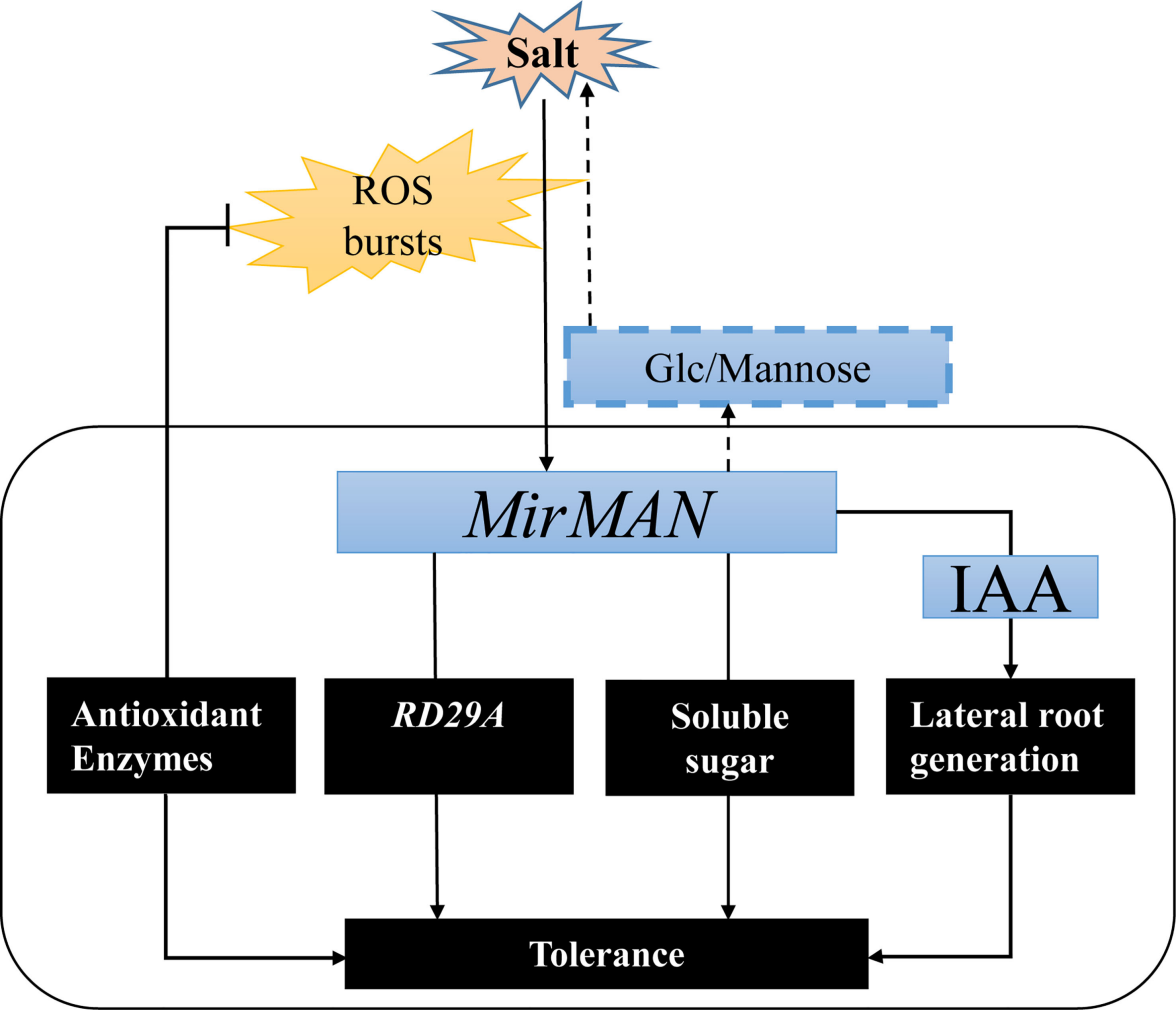
A hypothetical model showing the roles of MirMAN in regulating salt tolerance. MirMAN affects the expression of its downstream stress genes such as *RD29A*, mediating plant tolerance during development and salt stress. MirMAN can hydrolyze mannans and increase soluble sugar content to alleviate osmotic stress. It can also regulate plant tolerance by causing changes in the antioxidant enzyme system.

In summary, we successfully isolated *MirMAN* gene from *M. jalapa* and defined its location and function (**Figure 9**). The heterologous expression of *MirMAN* enhanced *Arabidopsis* growth and development and improved the resistance of transgenic *Arabidopsis* to salt stress by increasing soluble sugar contents and enhancing antioxidant defenses. *MirMAN* plays an important role in plants by enhancing root growth. It can be a candidate gene for plant molecular breeding and provide valuable insights into the physiological significance of *MAN* in plant abiotic stress.

## Data availability statement

The original contributions presented in the study are included in the article/**Supplementary Materials**, further inquiries can be directed to the corresponding author/s.

## Author contributions

MZ and HX were responsible for the study design. JX, CY, SC and HL drafted the manuscript. JX, CY and SJ performed the experiments. SJ, CY, HM and JL performed the statistical analyses. All authors contributed to the article and approved the submitted version.
