# Supplementary material for: Heterologous expression of MirMAN enhances root development and salt tolerance in Arabidopsis
Source: Front Plant Sci. 2023 Apr 14;14:1118548. doi: 10.3389/fpls.2023.1118548 (PMC10145921; doi:10.3389/fpls.2023.1118548)
Supplement: Supplementary file 1 [file DataSheet_1.pdf]

Supplementary Material

1 Supplementary Figures and Tables

1.1 Supplementary Figures

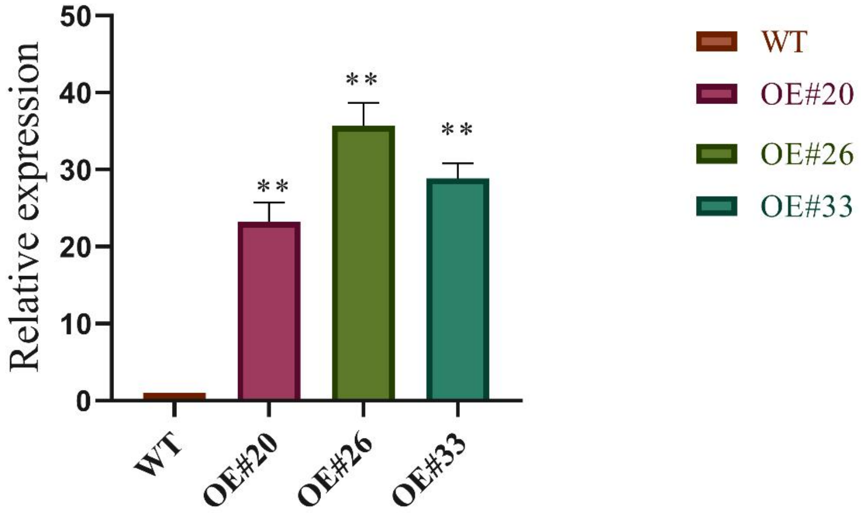

Supplementary Figure 1. Relative expression of *MirMAN* in different transgenic lines.

Data are means ± SD (n = 10). *$P < 0.05$, **$P < 0.01$, ***$P < 0.001$, Independent-Samples T Test.

A B

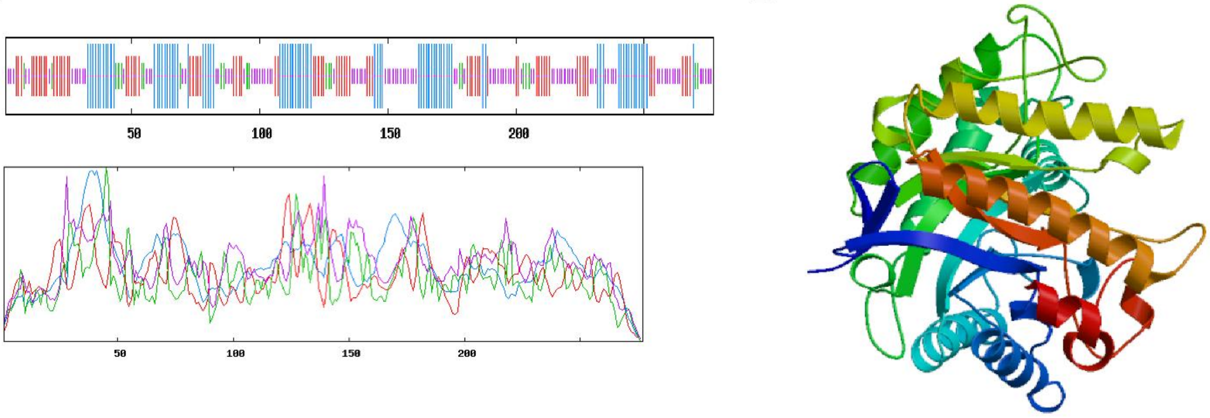

Supplementary Figure 2. Predicted structure of MirMAN.
A, Secondary structure of MirMAN.

Blue, alpha helix; green, beta turn; red, extended strand; purple, random coil. B, Predicted tertiary structure of MirMAN.

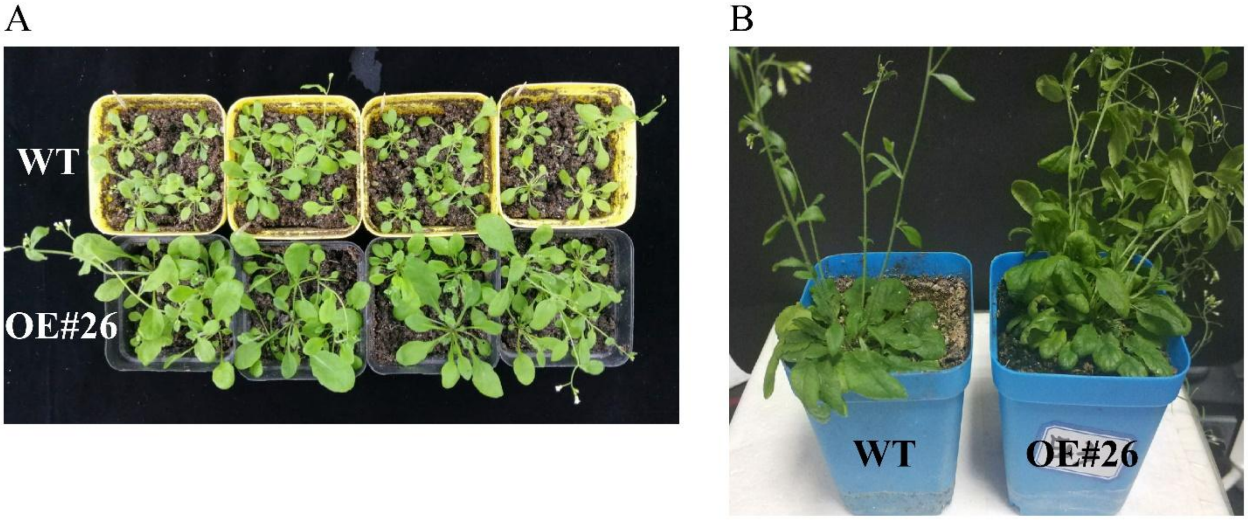

Supplementary Figure 3. The phenotypic observation of WT and OE#26 *Arabidopsis*. They were cultured for 6 weeks. WT, wild type.

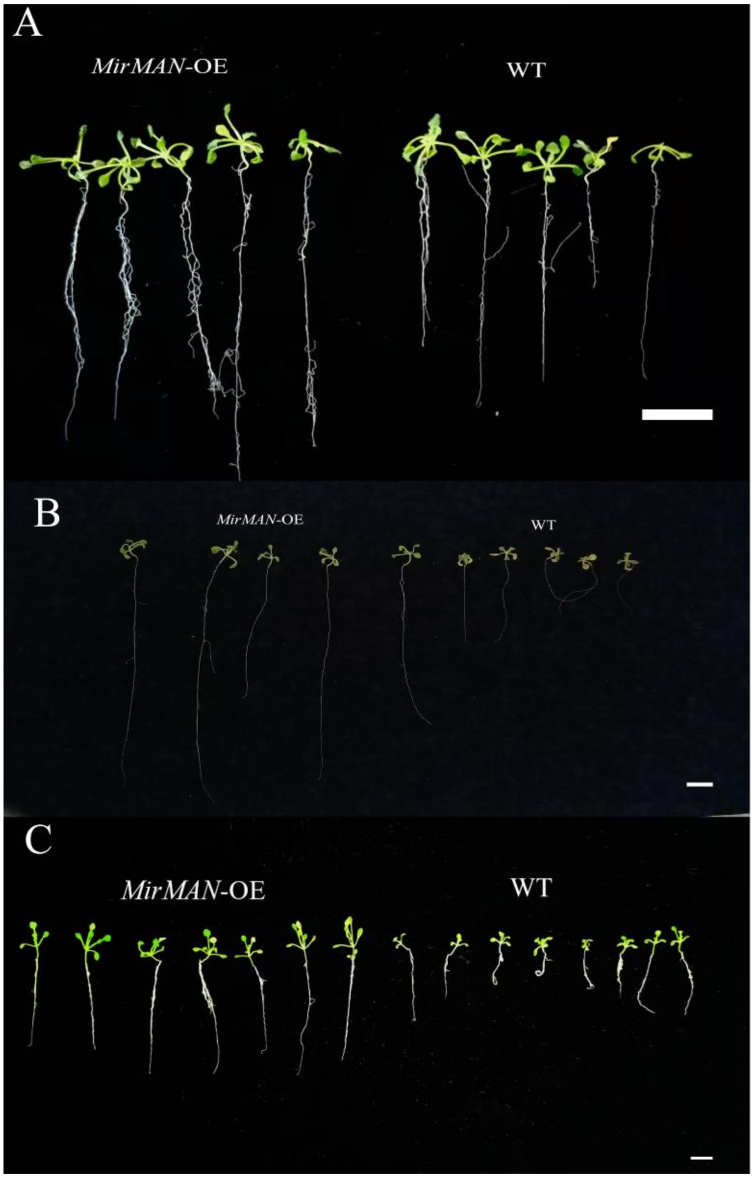

Supplementary Figure 4. Root growth of MirMAN-OE#26 and WT Arabidopsis on different concentrations of NaCl after 5 d.

Root growth under 100 mM, 150 mM, and 200 mM NaCl for 5 d of treatment (A-C). A,100 mM NaCl treatment. B, 150 mM NaCl treatment. C, 200 mM NaCl treatment. The 7-d-old seedings were transferred onto 0.5x MS agar medium with different concentrations NaCl for 5 d. Scale bar: 1 cm.

1.2 Supplementary Tables

Supplementary table 1. Gene-specific primers used for gene expression analysis by quantitative real-time PCR.

gene	Primers (5’-3’)
RD29A-F	ATTCACCATCCAGAAGAAGAGCATC
RD29A-R	ACTTCTGGGTCTTGCTCGTCA
ACTIN2-F	TGTGCCAATCTACGAGGGTTT
ACTIN2-R	TTTCCCGCTCTGCTGTTGT
MirMAN-F	GAAGATCTAAAGAAAGAAAAAATGAAAATAAAT
MirMAN-R	CGGACTAGTTCACCTCCTTAACTTTCTAATAATCCTA
MirMAN-RTF	TCAACACCTGACAAGAAGCGACA
MirMAN-RTR	CGGGGTAGGAGTGGACAGTAGC
